# Recurrent Multiple Evanescent White Dot Syndrome (MEWDS) Following First Dose and Booster of the mRNA-1273 COVID-19 Vaccine: Case Report and Review of Literature

**DOI:** 10.3390/vaccines10111776

**Published:** 2022-10-22

**Authors:** Matias Soifer, Nam V. Nguyen, Ryan Leite, Josh Fernandes, Shilpa Kodati

**Affiliations:** 1 National Eye Institute, National Institutes of Health, Bethesda, MD 20892, USA; 2 College of Medicine, University of Nebraska Medical Center, Omaha, NE 68198, USA; 3 School of Medicine, Georgetown University, Washington, DC 20007, USA; 4 DC Retina, Silver Spring, MD 20902, USA

**Keywords:** white dot syndromes, MEWDS, COVID-19 vaccine, mRNA vaccine

## Abstract

To report a rare case of a patient with two recurrent episodes of Multiple Evanescent White Dot Syndrome (MEWDS) associated with the second dose and second booster of the mRNA-1273 COVID-19 vaccine (Moderna), and to perform a literature review on COVID-19-vaccine-associated MEWDS. **Case Report:** A 31-year-old female was evaluated for a temporal scotoma and photopsias that started two weeks after the second dose of the Moderna COVID-19 vaccine. Dilated fundus findings were remarkable for unilateral, small whitish-yellow dots scattered around posterior pole of the left eye, consistent with a diagnosis of MEWDS. The symptoms resolved three months later without treatment. Approximately one year after the first vaccine, the patient received the second Moderna COVID-19 vaccine booster and experienced a recurrence of symptoms with an enlarged scotoma and similar examination findings. The patient was treated with a course of systemic corticosteroids with subsequent clinical improvement. **Conclusion:** Although uveitis following COVID-19 vaccines is rare, our case highlights a need for increased awareness amongst practitioners regarding COVID-19-vaccine-associated onset or recurrence of ocular inflammatory diseases.

## 1. Introduction

The white dot syndromes (WDS) refer to a group of ocular diseases that characteristically involve the outer retina and/or choroid and present with white-yellow lesions of varying morphologies on fundus examination. Amongst them, multiple evanescent white dot syndrome (MEWDS) has distinctive features, characterized by small spots at the level of the outer retina or RPE and is more commonly unilateral. This disease is usually self-limiting and occurs largely in healthy young adult females [1,2].

Although the pathogenesis of MEWDS remains poorly understood, different theories implicate a genetic and autoimmune predisposition contributing to the development of this disease. Interestingly, its onset has been associated with different viral vaccines including hepatitis A and B, human papillomavirus, influenza, measles-mumps-rubella, varicella virus, yellow fever, and most recently COVID-19 vaccines [3].

The Food and Drug Administration (FDA) authorized the messenger RNA (mRNA) vaccines from Moderna and Pfizer-BioNTech for SARS-CoV-2 virus (COVID-19) disease for emergency use in December 2020 [3]. Since then, other vaccines with different compositions have been widely utilized around the world, and the efforts have reduced the morbidity and mortality effectively caused by the COVID-19 virus [4,5]. There have been approximately 600 million COVID-19 infections with 6.5 million associated deaths to date worldwide. The number of vaccinations has now exceeded 12.5 billion, and the incidences of infections and death have decreased significantly since January 2022 [6]. The positive global effect that these vaccines have had is unquestionable. Nevertheless, reports of very rare side effects including ophthalmic findings have been noted with their use [3,7].

Herein, we report a case of recurrent MEWDS associated with the mRNA-1273 COVID-19 vaccine. Furthermore, we performed a literature review on COVID-19-vaccine-associated cases of MEWDS reported until 20 July 2022, using PubMed and Google scholar search engines for terms “multiple evanescent white dot syndrome, MEWDS, uveitis, and COVID-19 vaccine”.

## 2. Case Report

A 31-year-old white female was evaluated for a new onset temporal scotoma and photopsias, 14 days after the second dose of the mRNA-1273 COVID-19 vaccine (Moderna). She was seen by an outside ophthalmologist, and her fundus examination was remarkable for multifocal, outer retinal dots scattered around the posterior pole in the left eye consistent with a diagnosis of MEWDS. The patient was observed without any intervention, and her symptoms resolved within 3 months. Approximately 12 months after the second dose, the patient received the second booster dose and experienced a recurrence of symptoms, which prompted referral to our clinic.

**Figure 1:**
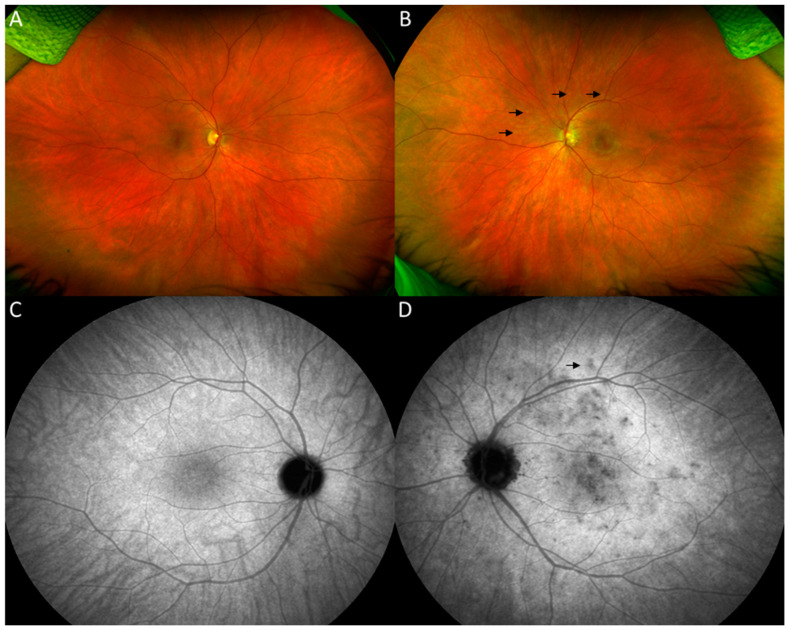
Optos ultrawide-field fundus images of the right eye showing normal fundus (**A**). The left eye demonstrates a mottled appearance of the macula and areas of multiple scattered hypopigmented spots in the posterior pole (dark arrows) (**B**). Late-phase indocyanine green angiography (ICG) of the left eye shows multiple spots of hypocyanescence in the posterior pole, some of which correspond to the spots on color fundus photos (**D**). Late-phase ICG of the right eye (**C**) was unremarkable.

**Figure 2:**
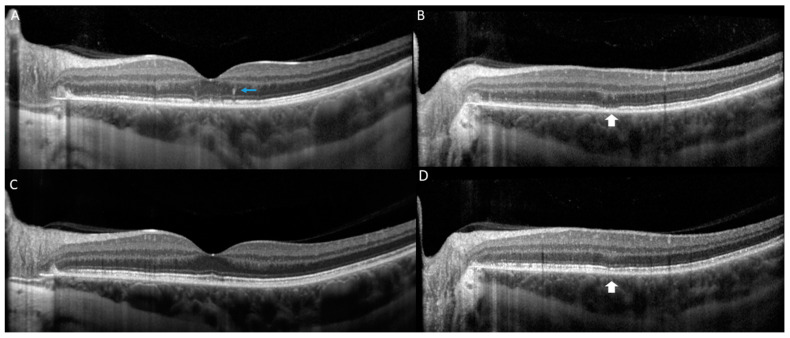
Optical coherence tomography (OCT) of the left eye at the initial presentation is notable for presence of hyperreflective dots in outer retina (blue arrow) and multifocal loss of the ellipsoid zone (EZ) (white arrow) (**A**,**B**). At 1 month follow up, reconstitution of the EZ (white arrow) is observed and improvement in outer retinal hyperreflectivity (**C**,**D**).

**Figure 3:**
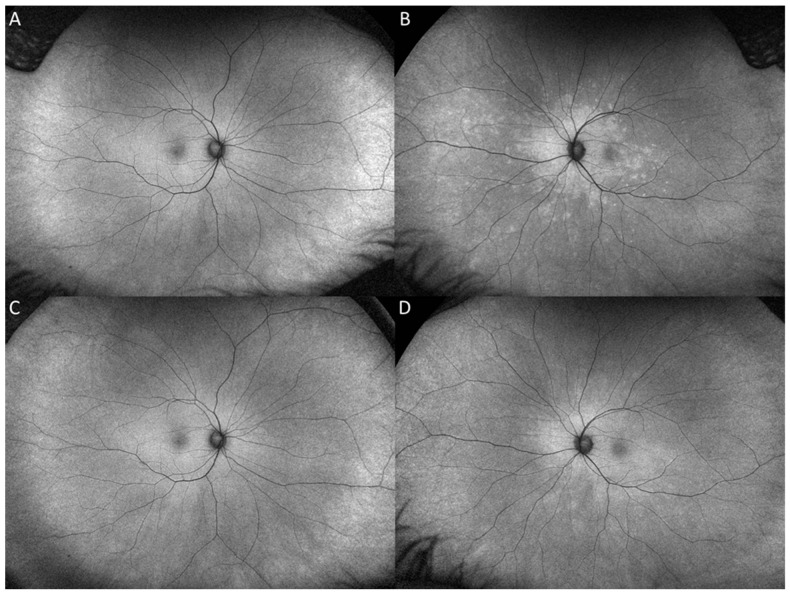
Fundus autofluorescence (FAF) at presentation of the left eye at presentation demonstrates a confluent area of hyperautofluorescence surrounding the optic nerve and posterior pole with hyperautofluorescent dots extending to the temporal macula and mid nasal periphery (**B**) FAF at 1 month follow-up demonstrates decreased hyperautofluoresence and ill-defined borders. (**D**)**.** FAF of the right eye at presentation and final visit are unremarkable (**A**,**C**).

On our evaluation, the patient reported an enlarged scotoma in her left eye with increased photopsias. Past medical history was unremarkable, except for a remote episode of Lyme disease, which was successfully treated with doxycycline. Past ocular history was significant for myopia, and she was status-post myopic LASIK surgery in both eyes in 2019. On examination, best-corrected visual acuity (BCVA) was 20/16 on the right eye (OD) and 20/50 on the left eye (OS), and intraocular pressure (IOP) was 13 mm Hg in both eyes (OU). Slit-lamp examination revealed a quiet anterior chamber in both eyes and trace pigmented anterior vitreous cells in the left eye. On dilated fundus examination, a few scattered faint hypopigmented round dots of differing sizes, especially nasal to the optic nerve (Figure 1B) were noted. Optical Coherence Tomography (OCT) of the left eye revealed multifocal areas of ellipsoid zone (EZ) loss with associated areas of outer retinal hyperreflectivity (Figure 2A–C). Fundus autofluorescence (FAF) of the left eye revealed a confluent area of hyperautofluorescence in the posterior pole with scattered hyperautofluorescent dots (Figure 3B). Fluorescein angiography (FA) of the left eye demonstrated hyperfluorescent staining of the corresponding areas. Late-phase indocyanine green (ICG) imaging showed diffuse hypocyanescent spots in the posterior pole (Figure 1D). The clinical examination and imaging studies of the right eye were unremarkable. A uveitic laboratory work-up was performed, which was unrevealing for QuantiFERON, syphilis IgG antibody and angiotensin converting enzyme. The clinical presentation, examination, and imaging findings were thought to be consistent with MEWDS. After a discussion with the patient, a course of 60 mg of oral prednisone tapered over 8 weeks was started. The patient returned for follow-up two weeks later with improvement in symptoms and BCVA OS had improved to 20/25 OS. Four-weeks after the initiation of prednisone, BCVA OS remained stable at 20/25. OCT revealed reconstitution of the EZ and a decrease in outer retinal hyperreflectivity (Figure 2C,D), and FAF demonstrated a reduction in hyperautofluoresence (Figure 3D).

## 3. Discussion

We report a de novo case of MEWDS following the second dose of COVID-19 mRNA-vaccination and a subsequent recurrence after the second booster. To the best of our knowledge, this is the second case of COVID-19-vaccine-associated MEWDS which developed a recurrent course, with both episodes temporally associated with vaccine doses. Recurrences are uncommonly reported in MEWDS. A large retrospective cohort of 111 patients with MEWDS, observed recurrent disease in only 14% of cases [8] Although our patient had improved symptoms after the initiation of corticosteroids, the efficacy of treatment with systemic corticosteroids for MEWDS remains unclear. In a study of 51 patients with MEWDS, those who received systemic prednisone did not achieve a better final BCVA than those who did not; however, lower baseline BCVA at presentation and younger age were predictive of worse outcomes [9]. Although the use of corticosteroids with this subpopulation is reasonable, further studies are needed to determine the efficacy of corticosteroids in MEWDS.

The association between COVID-19 vaccines and MEWDS is yet to be elucidated. The causality assessment of an adverse event following immunization (AEFI) provides criteria to assess causality between a vaccine and an adverse event [10]. In our case, factors supporting a causal association are that this presentation occurred in an otherwise healthy patient, without a previous history of uveitis, and was temporally associated, as the doses of the vaccine preceded the onset of disease within the “plausible” time window on both occasions. Notably, the patient denied a viral prodrome, which is commonly associated with MEWDS. Finally, MEWDS has been reported in association with different vaccines [11], as well as more recently, COVID-19 immunizations (Table 1).

To date, 13 cases of COVID-19-vaccine-associated MEWDS have been reported to date in the literature (Table 1). The average patient age was 39.5 years (ranging from 15 to 71 years old) with a female predominance (9:4), and the only reported races (5/13) were Asian (3) or White (2). Notably, these demographics and clinical features are similar to non-vaccine related MEWDS [2]. The most common vaccine manufacturer was Pfizer-BioNTech (9/13) followed by CoronaVac (2/13), Medigen (1/13), and Moderna (1/13). Of note, approximately 360 million Pfizer doses have been administered worldwide compared to Moderna’s 229 million [12], which may bias the data towards a greater association between the Pfizer vaccine and MEWDS than with the Moderna vaccine or others. From our review, six cases developed symptoms after the first dose, seven patients after the second dose, and one after the first booster. Interestingly, the large majority of cases (12/13) had their first onset of MEWDS following the vaccine. One case had a previous episode of MEWDS nine years prior and experienced recurrence with the vaccine [13]. Of note, one case developed a new episode following the first dose, then a recurrence of MEWDS in the same eye following the second vaccine [13]. Nine patients received no treatment, and four patients received a short course of systemic corticosteroids. Nearly all patients had complete resolution of visual symptoms, with majority having 20/20 as their final visual acuity [13].

These findings are highly comparable with those of other reported vaccine-associated MEWDS presentations. Ng et al. reviewed eight cases of vaccine-associated MEWDS, which included immunizations against rabies, HPV, hepatitis A and B, meningococcal, yellow fever, and influenza [11]. Patients had an average age of 31.7 years (ranging from 16 to 53) with female predominance (2:1), and the disclosed racial data indicated that only White (44.4%) and Asian (22.2%) races were affected; however, this association should not be restricted to these ethnic populations. Overall, patients had significant visual recovery, although one reported a gradual loss of peripheral vision for 2 years following MEWDS [11]. Altogether, MEWDS associated with the COVID-19 vaccines and others, typically occurs in young to middle age healthy females, has a good prognosis and mostly monophasic course of disease.

The Pfizer BNT and Moderna COVID-19 vaccines are both mRNA vaccines and received their authorizations almost simultaneously in December 2020 (FDA and UK). This vaccine encapsulates the mRNA in lipid nanoparticles (LNP) that encode for the S protein of SARS-CoV-2 [14]. Unlike most vaccines, the mRNA molecules function as both the antigen and the adjuvant; thus, it can avoid the need for added molecules that may induce toxicity. Although the exact mechanisms by which the mRNA COVID-19 vaccines cause uveitis is still unclear, it has been proposed that these vaccines can activate type I interferon (IFN) proinflammatory cascades which can stimulate autoimmunity in predisposed individuals [15,16]. The inoculated mRNA may activate intracellular RNA-sensors, namely endosomal Toll-like receptors, with a subsequent increase in IFN production which may stimulate autoimmunity [17]. However, although we are reporting an association of MEWDS with the mRNA COVID-19 Moderna vaccine, the underlying pathogenesis is likely due to a shared etiological mechanism across different vaccines, given the reports of MEWDS following COVID-19 non-mRNA vaccines as well as non-COVID -19 vaccines. Many theories have been proposed to explain this phenomenon which include molecular mimicry [18], hypersensitivity reactions [19], and autoimmunity induced by adjuvants (ASIA) [20]. Future work is needed to better understand the multifactorial risk factors including genetic and environmental associations that predispose certain individuals to the development of MEWDS.

COVID-19 vaccines have been associated with multiple ocular inflammatory diseases including anterior uveitis [21], acute macular neuroretinopathy, bilateral acute zonal occult outer retinopathy (AZOOR) [15], Vogt–Koyanagi–Harada [22], optic neuropathies [23] and corneal graft rejection [24,25]. It is important to note that the uveitis following COVID-19 vaccine have a good prognosis as shown by multinational case series of 70 patients with onset of ocular inflammatory diseases associated with different COVID-19 vaccines [3]. In this series, the majority of patients presented with anterior uveitis (58.6%), followed by posterior uveitis and scleritis. Most of these were not severe and their course was notable for unchanged final visual acuity in 93% of cases. The majority were either observed without treatment or received topical corticosteroids (70%) [3]. In our case and literature review, all patients improved with visual acuity greater than 20/40, and the majority improved to 20/20 (73%).

**Table 1:** Summary of cases of COVID-19-vaccine-associated MEWDS.

Authors	Age	Sex	Race	Vaccine	Dose	Time from Vaccine to Symptoms (Days)	Ocular Symptoms	Past History	Course	Initial VA	Final VA	Treatment	Resolution
Bolletta et al. [26]	53	M	_	Pfizer BNT162b2	2nd	28	Decreased VA, scotoma,	None	Acute	20/25	20/20	Observation	Complete resolution
	18	F	_	Pfizer BNT162b2	1st	4	Blurred vision, visual field defect	None	Acute	20/66	20/20	Observation	Complete resolution
	48	M	_	Pfizer BNT162b2	1st	7	Decreased VA	None	Acute	20/400	20/20	Observation	Complete resolution
Xu et al. [13]	49	F	_	Sinovac- CoronaVAC vaccine (Inactivated)	1st	2	Blurred vision	MEWDS	Recurrent	20/100	20/20	Tapered prednisone, starting with 20 mg	Complete resolution
Lin et al. [27]	36	F	Taiwanese	Medigen Vaccine Biologics Corporation (MVC) COVID-19 Vaccine	1st	2	Photopsia	High myopia (−9.75D/−7.5D)	Acute	20/25	20/20	observation	Complete resolution in 4 weeks
Smith et al. [28]	15	M	_	Pfizer BNT162b2	2nd	14	Blurred vision, myodesopsia, photopsia	None	Acute	20/100	20/20	Tapered oral prednisone, starting at 40 mg	Complete resolution in 2 weeks
	21	F	_	Pfizer BNT162b2	2nd	21	Blurred vision	None	Acute	20/60	20/20	Tapered oral prednisone, starting at 40 mg	Complete resolution in 2 weeks
Yasuda et al. [29]	67	F	Japanese	Pfizer BNT162b2	2nd	1	Scotoma, photopsia, blurred vision	None	Acute	20/100	20/25	_	Patient also developed moderate vitritis, almost complete resolution at 2 weeks.
Inagawa et al. [30]	30	F	Japanese	Pfizer BNT162b2	1st, 2nd	7 and 3, respectively	Blurred vision	None	Recurrent	20/20 OU	20/30	Topical 0.1% fluorometholone for 21 days	Both eyes had fundus abnormalities L>R
Tomishige et al. [31]	38	F	White	Sinovac- CoronaVac vaccine (Inactivated)	1st	7	Photopsia, decreased VA	None	Acute	20/400	20/20	Tapered oral prednisone, starting at 80 mg oral prednisone	Complete resolution in 4 weeks
Rabinovitch et al. [32]	28	F	_	Pfizer BNT162b2	2nd	0	Blurred vision, scotoma, photopsia	None	Acute	_	_	_	Significant improvement on FU
	39	M	_	Pfizer BNT162b2	2nd	_	Blurred vision, scotoma, photopsia	None	Acute	_	_	_	Significant improvement on FU
Alhabshan et al. [33]	71	F	White	Moderna mRNA-1273	1st booster	3	Blurred vision and scotoma	Myopic and retinal tear	Acute	20/30	_	_	Spontaneous improvement

## 4. Conclusions

In conclusion, we have reported a case of recurrent episodes of MEWDS following COVID-19 mRNA-1273 vaccines. The majority of vaccine-associated MEWDS episodes were previously in healthy, young females and resembled those of non-COVID-19-vaccine-associated MEWDS. Although the prognosis for this disease is favorable, physicians should be aware of this association so that these patients can be rapidly identified and offered prompt management and counseling.

## Data Availability

Not applicable.

## References

[1] Jampol L.M., Sieving P.A., Pugh D., Fishman G.A., Gilbert H. (1984). Multiple evanescent white dot syndrome. I. Clinical findings. Arch. Ophthalmol..

[2] Papasavvas I., Mantovani A., Tugal-Tutkun I., Herbort C.P. (2021). Multiple evanescent white dot syndrome (MEWDS): Update on practical appraisal, diagnosis and clinicopathology; a review and an alternative comprehensive perspective. J. Ophthalmic Inflamm. Infect..

[3] Testi I., Brandão-de-Resende C., Agrawal R., Pavesio C. (2022). Ocular inflammatory events following COVID-19 vaccination: A multinational case series. J. Ophthalmic Inflamm. Infect..

[4] Watson O.J., Barnsley G., Toor J., Hogan A.B., Winskill P., Ghani A.C. (2022). Global impact of the first year of COVID-19 vaccination: A mathematical modelling study. Lancet Infect. Dis..

[5] Sharif N., Alzahrani K.J., Ahmed S.N., Dey S.K. (2021). Efficacy, Immunogenicity and Safety of COVID-19 Vaccines: A Systematic Review and Meta-Analysis. Front. Immunol..

[6] WHO Coronavirus (COVID-19) Dashboard. https://covid19.who.int.

[7] Rosenblum H.G., Hadler S.C., Moulia D., Shimabukuro T.T., Su J.R., Tepper N.K., Ess K.C., Woo E.J., Mba-Jonas A., Alimchandani M. (2021). Use of COVID-19 Vaccines After Reports of Adverse Events Among Adult Recipients of Janssen (Johnson & Johnson) and mRNA COVID-19 Vaccines (Pfizer-BioNTech and Moderna): Update from the Advisory Committee on Immunization Practices—United States, July 2021. MMWR Morb. Mortal. Wkly. Rep..

[8] Ramakrishnan M.S., Patel A.P., Melles R., Vora R.A. (2021). Multiple Evanescent White Dot Syndrome: Findings from a Large Northern California Cohort. Ophthalmol. Retina..

[9] Bosello F., Westcott M., Casalino G., Agorogiannis G., Micciolo R., Rees A., Pavesio C. (2022). Multiple evanescent white dot syndrome: Clinical course and factors influencing visual acuity recovery. Br. J. Ophthalmol..

[10] WHO (2019). Causality Assessment of an Adverse Event Following Immunization (AEFI): User Manual for the Revised WHO Classification.

[11] Ng C.C., Jumper J.M., Cunningham E.T. (2020). Multiple evanescent white dot syndrome following influenza immunization—A multimodal imaging study. Am. J. Ophthalmol. Case Rep..

[12] COVID Vaccinations Administered Number by Manufacturer U.S. 2022. Statista. https://www.statista.com/statistics/1198516/covid-19-vaccinations-administered-us-by-company/.

[13] Chen Y., Xu Z., Wang P., Li X.M., Shuai Z.W., Ye D.Q., Pan H.F. (2022). New-onset autoimmune phenomena post-COVID-19 vaccination. Immunology.

[14] Yadav T., Srivastava N., Mishra G., Dhama K., Kumar S., Puri B., Saxena S.K. (2020). Recombinant vaccines for COVID-19. Hum. Vaccines Immunother..

[15] Maleki A., Look-Why S., Manhapra A., Foster C.S. (2021). COVID-19 Recombinant mRNA Vaccines and Serious Ocular Inflammatory Side Effects: Real or Coincidence?. J. Ophthalmic Vis. Res..

[16] Ngo S.T., Steyn F.J., McCombe P.A. (2014). Gender differences in autoimmune disease. Front. Neuroendocrinol..

[17] Choubey D., Moudgil K.D. (2011). Interferons in Autoimmune and Inflammatory Diseases: Regulation and Roles. J. Interferon Cytokine Res..

[18] Vojdani A., Kharrazian D. (2020). Potential antigenic cross-reactivity between SARS-CoV-2 and human tissue with a possible link to an increase in autoimmune diseases. Clin. Immunol. Orlando Fla..

[19] Sawant O.B., Wright S.S.E., Jones K.M., Titus M.S., Dennis E., Hicks E., Majmudar P.A., Kumar A., Mian S.I. (2021). Prevalence of SARS-CoV-2 in human post-mortem ocular tissues. Ocul. Surf..

[20] Bragazzi N.L., Hejly A., Watad A., Adawi M., Amital H., Shoenfeld Y. (2020). ASIA syndrome and endocrine autoimmune disorders. Best Pract. Res. Clin. Endocrinol. Metab..

[21] Rabinovitch T., Ben-Arie-Weintrob Y., Hareuveni-Blum T., Shaer B., Vishnevskia-Dai V., Shulman S., Newman H., Biadsy M., Masarwa D., Fischer N. (2021). Uveitis after the BNT162b2 mRNA vaccination against SARS-CoV-2 Infection: A Possible Association. Retina Phila Pa.

[22] Haseeb A.A., Solyman O., Abushanab M.M., Abo Obaia A.S., Elhusseiny A.M. (2022). Ocular Complications Following Vaccination for COVID-19: A One-Year Retrospective. Vaccines.

[23] Elnahry A.G., Asal Z.B., Shaikh N., Dennett K., Elmohsen M.N.A., Elnahry G.A., Shehab A., Vytopil M., Ghaffari L., Athappilly G.K. (2021). Optic neuropathy after COVID-19 vaccination: A report of two cases. Int. J. Neurosci..

[24] Nioi M., d’Aloja E., Fossarello M., Napoli P.E. (2021). Dual Corneal-Graft Rejection after mRNA Vaccine (BNT162b2) for COVID-19 during the First Six Months of Follow-Up: Case Report, State of the Art and Ethical Concerns. Vaccines.

[25] Jin S.X., Juthani V.V. (2021). Acute Corneal Endothelial Graft Rejection With Coinciding COVID-19 Infection. Cornea.

[26] Bolletta E., Iannetta D., Mastrofilippo V., Simone L.D., Gozzi F., Croci S., Bonacini M., Belloni L., Zerbini A., Adani C. (2021). Uveitis and Other Ocular Complications Following COVID-19 Vaccination. J. Clin. Med..

[27] Multiple Evanescent White Dot Syndrome Following Medigen Vaccine Biologics Corporation COVID-19 Vaccination—PubMed. https://pubmed.ncbi.nlm.nih.gov/35442848/.

[28] Multiple Evanescent White Dot Syndrome following COVID-19 mRNA Vaccination in Two Patients—PubMed. https://pubmed.ncbi.nlm.nih.gov/35201960/.

[29] Yasuda E., Matsumiya W., Maeda Y., Kusuhara S., Nguyen Q.D., Nakamura M., Hara R. (2022). Multiple evanescent white dot syndrome following BNT162b2 mRNA COVID-19 vaccination. Am. J. Ophthalmol. Case Rep..

[30] Inagawa S., Onda M., Miyase T., Murase S., Murase H., Mochizuki K., Sakaguchi H. (2022). Multiple evanescent white dot syndrome following vaccination for COVID-19: A case report. Medicine.

[31] Tomishige K.S., Novais E.A., Finamor L.P.D.S., Nascimento H.M.d., Belfort R. (2022). Multiple evanescent white dot syndrome (MEWDS) following inactivated COVID-19 vaccination (Sinovac-CoronaVac). Arq. Bras. Oftalmol..

[32] Uveitis after the BNT162b2 mRNA Vaccination against SARS-CoV-2 Infection: A Possible Association—PubMed. https://pubmed.ncbi.nlm.nih.gov/34369440/.

[33] Alhabshan R., Scales D. (2022). Multiple Evanescent White Dot Syndrome Developing Three Days following Administration of mRNA-1273 Booster Vaccine: Case Report. Case Rep. Ophthalmol..

